# Delivering a national de-adoption programme: a documentary analysis of local commissioning policy compliance with England's Evidence-based Interventions programme (EBI)

**DOI:** 10.1186/s12913-025-13012-0

**Published:** 2025-07-29

**Authors:** Carmel Conefrey, Nicola Farrar, Maeve Coyle, Mike Bell, Jane Blazeby, Christopher Burton, Jenny Donovan, Andy Gibson, Joel Glynn, Tim Jones, Angus McNair, Josie Morley, Amanda Owen-Smith, Ellen Rule, Gail Thornton, Victoria Tucker, Iestyn Williams, William Hollingworth, Leila Rooshenas

**Affiliations:** 1 Population Health Sciences, Bristol Medical School, University of Bristol, Canynge Hall, 39 Whatley Road, Bristol, BS8 2PS UK; 2 NIHR Bristol Biomedical Research Centre, University Hospitals Bristol and Weston NHS Foundation Trust and University of Bristol, Bristol, UK; 3 National Institute for Health and Care Research Applied Research Collaboration West (NIHR ARC West) at University Hospitals Bristol and Weston NHS Foundation Trust, Bristol, UK; 4 Bristol Centre for Surgical Research, Bristol Medical School, Population Health Sciences, University of Bristol, Bristol, UK; 5 School of Allied and Public Health Professions, Canterbury Christ Church University, Canterbury, UK; 6 Musculoskeletal Research Unit, Translational Health Sciences, Bristol Medical School, Southmead Hospital, Bristol, UK; 7 North Bristol NHS Trust, Bristol, UK; 8 Gloucestershire Integrated Care Board (ICB), Brockworth, UK; 9 Public Contributor, Bristol, UK; 10 Bristol, North Somerset and South Gloucestershire Integrated Care Board (ICB), Bristol, UK; 11 Health Services Management Centre, University of Birmingham, Birmingham, UK

**Keywords:** De-adoption, Health policies, Commissioning, Surgical procedures, Documentary analysis

## Abstract

**Background:**

In 2019 the English National Health Service (NHS) launched a national de-adoption programme to stop or limit access to surgical procedures considered to have little, or uncertain, evidence of benefit to justify their risks and/or costs: the Evidence-Based Interventions (EBI) programme. Central to the programme was the publication of guidance detailing clinical recommendations targeting 17 surgical procedures: four to be stopped and 13 to be restricted to patients satisfying specific criteria. Local commissioning organisations, NHS bodies responsible for purchasing surgical services, were instructed to reflect national EBI recommendations in their local commissioning policies. This study (which is part of the NIHR OLIVIA study, an evaluation of the EBI programme) assessed local commissioning policy compliance with EBI recommendations and identified funding mechanisms employed locally to promote enforcement.

**Methods:**

A documentary analysis was conducted on a purposive sample of local commissioning policies for each of the 17 EBI surgical procedures. Local policies were compared to EBI recommendations and any differences were categorised against an established five category framework for capturing differences in local policies. Funding mechanisms were also recorded. Data were analysed using descriptive statistics supported by written summaries to describe the nature of discrepancies between local and national recommendations.

**Results:**

Three hundred six local commissioning policies were analysed. 72% (44/61) of procedures to be stopped and 43% (106/245) of restricted access policies matched EBI recommendations. Concordance rates varied by surgical procedures. Where local policies for the 13 restricted access procedures differed, variations were most commonly categorised as differences in diagnostic approach followed by differences in specification of symptom severity and disease progression. The funding mechanism most frequently stated for the stopped procedures was ‘Individual Funding Request’ (74%, 45/61), whilst for restricted access procedures, policies relied on ‘Criteria Based Access’ (48%, 117/245) followed by ‘Prior Approval’ (33%, 80/245).

**Conclusion:**

This study, to our knowledge, is the first to explore variation between local and national de-adoption policies. With under half of local commissioning policies matching national EBI recommendations, reliance on the take up of national de-adoption policy is inadequate. More support is needed for local commissioners to reflect national guidance.

**Supplementary Information:**

The online version contains supplementary material available at 10.1186/s12913-025-13012-0.

## Background

In recent years, growing attention has been paid to how best to reduce provision of surgical procedures that have little, or uncertain, evidence of benefit to justify their risks and/or costs. Such initiatives are variously referred to as ‘disinvestment’ [1], ‘de-adoption’ [2] and ‘de-implementation’ [3]. Despite broad international appeal [4–7], the ability of de-adoption (the term used in this study) initiatives to reduce surgical activity is thus far limited [8–11].

De-adoption is widely considered a complex process [12–14]. Elements of complexity arise from the plethora of stakeholders that need to engage for de-adoption to happen [15] combined with the governance of de-adoption [13]. Many interventions are at local [16] and regional levels [17]. Whilst there are examples of programmes promoted nationally, the highest profile being Choosing Wisely [18, 19], there are only a few examples of programmes that are initiated and steered by national government. One example is in Sweden where the health system is largely publicly funded and is operationalised through 21 regions. In a study of the country’s approach, Augusston et al. [20] reported that de-adoption was governed through four mechanisms: health technology assessment, national clinical practice recommendations, control over pharmaceutical products and a national system for knowledge management.

In April 2019, the National Health Service (NHS) in England launched the Evidence-Based Interventions programme (EBI) in partnership with the Academy of Medical Royal Colleges [21]. Leadership by the publicly-funded NHS signalled a top-down approach to de-adoption and followed on from earlier embryonic attempts to de-adopt via ‘do not do’ lists of recommendations published by the National Institute for Health and Care Excellence (NICE) a decade earlier [14]. The EBI programme aimed to reduce inappropriate surgical activity and avoidable harm to patients, thus freeing up staff and financial resource to nurture innovation. In addition, the programme aspired to more standardised local commissioning policies and a reduction in unwarranted geographical variation [21].

A leading mechanism for implementing the EBI programme was the publication of guidance containing sets of recommendations determining the use of surgical procedures across multiple specialties [21]. The first set of guidance published concerned 17 procedures, and with later guidance releases, now cover up to 60 surgical procedures and diagnostic tests. This study concerns the initial tranche of 17 recommendations which were divided into two categories. Category 1 comprised four procedures to be stopped except in exceptional circumstances, often referred to as ‘do not do’ procedures. Category 2 comprised 13 procedures that could be commissioned and performed when specific threshold criteria were met, referred to as ‘restricted access’ procedures. For example, criteria might include attempts to manage the condition with more conservative options, or the requirement for disease severity thresholds to be met before surgical referral. The EBI programme stated that the recommendations drew on pre-existing clinical criteria from NICE Guidance and specialist societies [21].

The 2019 EBI Guidance was primarily aimed at Clinical Commissioning Groups (CCGs), and at that time, England was geographically divided into 106 CCGs. These were NHS bodies responsible for assessing, prioritising, and purchasing services to meet the needs of their local population. In 2022, the commissioning architecture was reconfigured and 42 Integrated Care Boards (ICBs), new NHS bodies, replaced CCGs (hereafter the term ‘commissioning organisations’ is used). Commissioning organisations are funded by and accountable to NHS England to commission elective hospital care - the focus of the EBI programme – and also mental health services, urgent and emergency care, primary care and medicines and community care. In 2021/2, nearly 70% of NHS England’s budget was allocated to commissioning organisations [22] for these services. The funding per commissioning organisation is determined by population size and adjusted upwards to account for additional local costs, associated for example with a high proportion of older people, or high service delivery costs. Whilst commissioning organisations have much autonomy over how they use funding to meet local needs, they must also deliver on national health policy priorities [23, 24].

The EBI guidance was statutory, which meant that commissioning organisations were legally required to ‘have regard to it’ when developing their local commissioning policies. Local commissioning policies are public documents informing general practitioners, secondary care providers and the public about what surgical procedures will be funded by the NHS locally and the clinical criteria that patients need to meet to be eligible for consideration for a procedure. The leverage of the national EBI Guidance was strengthened further by its inclusion in the NHS Standard Contract, the contractual document that commissioning organisations use when commissioning services from healthcare providers [25].

To promote delivery of the EBI programme, the Guidance referred to payment mechanisms to be used between commissioning organisations and healthcare providers. For the four ’do not do’ procedures, costs were only to be reimbursed where a clinician successfully demonstrated that a patient had exceptional circumstances which warranted the use of the procedure. Payment in those instances was managed by commissioning organisations through a mechanism called the Individual Funding Request (IFR). Where a procedure was performed without securing an IFR in advance, zero payment was to be made to the healthcare provider. For the remaining 13 procedures, the EBI Guidance was less prescriptive, leaving commissioning organisations to choose a payment mechanism that ensured compliance. Typical mechanisms included ‘Criteria Based Access’ (CBA) and ‘Prior Approval’ (PA). Both required a healthcare professional (HCP) to assess a patient’s eligibility against the relevant ‘threshold criteria’. Where a commissioning organisation managed payment for a procedure with CBA, provided the patient satisfied the threshold criteria, a HCP could proceed with a procedure on the assumption that it would be funded. Where a procedure was managed with PA, in addition to demonstrating compliance with the criteria, the HCP needed to secure approval for payment from the local commissioning organisation in advance of performing the procedure.

The aim of this study was firstly, to assess local policy compliance with national de-adoption recommendations, and secondly, to identify the funding mechanisms employed by local commissioning organisations to promote policy enforcement. This work forms part of the ‘OLIVIA’ study investigating the delivery, impact and acceptability of a national de-adoption intervention (the EBI programme), with a view to producing evidence-informed recommendations to guide future de-adoption efforts [26].

## Method

### Design

This was a documentary analysis of NHS commissioning policies for 17 surgical procedures featured in the 2019 EBI programme. Analysis was informed by the READ approach which involves four sequential steps (1) readying materials, (2) extracting data, (3) analysing data and (4) distilling findings [27]. The sub-sections below reflect the READ steps with some minor adjustments to nomenclature.

### Readying materials

There were 106 commissioning organisations, and assuming that each organisation had a policy for each of the 17 procedures, there were potentially 1,802 relevant policies from which to sample. The approach to sampling is detailed below.

#### Sampling commissioning organisations

Commissioning organisations were purposively sampled for each of the 17 procedures based on their progress towards reducing their surgical activity. Using EBI Dashboard [28] data, members of the research team (JG, TJ) identified surgical procedure rates in the year preceding introduction of the EBI programme (April 2018 – March 2019) and 12 months after its introduction (March 2019 – February 2020). For each surgical procedure, the 106 commissioning organisations were ranked according to the difference in absolute activity between the two time periods. The commissioning organisations were sampled by selecting the ‘top 10’ (i.e. with ‘most activity reduction’) and ‘bottom 10’ (i.e. with ‘least activity reduction’) to arrive at a sample of 20 commissioning organisations per procedure. The sampling process resulted in 17 distinct sets of commissioning organisations.

#### Sampling commissioning organisation policies

**Fig. 1:**
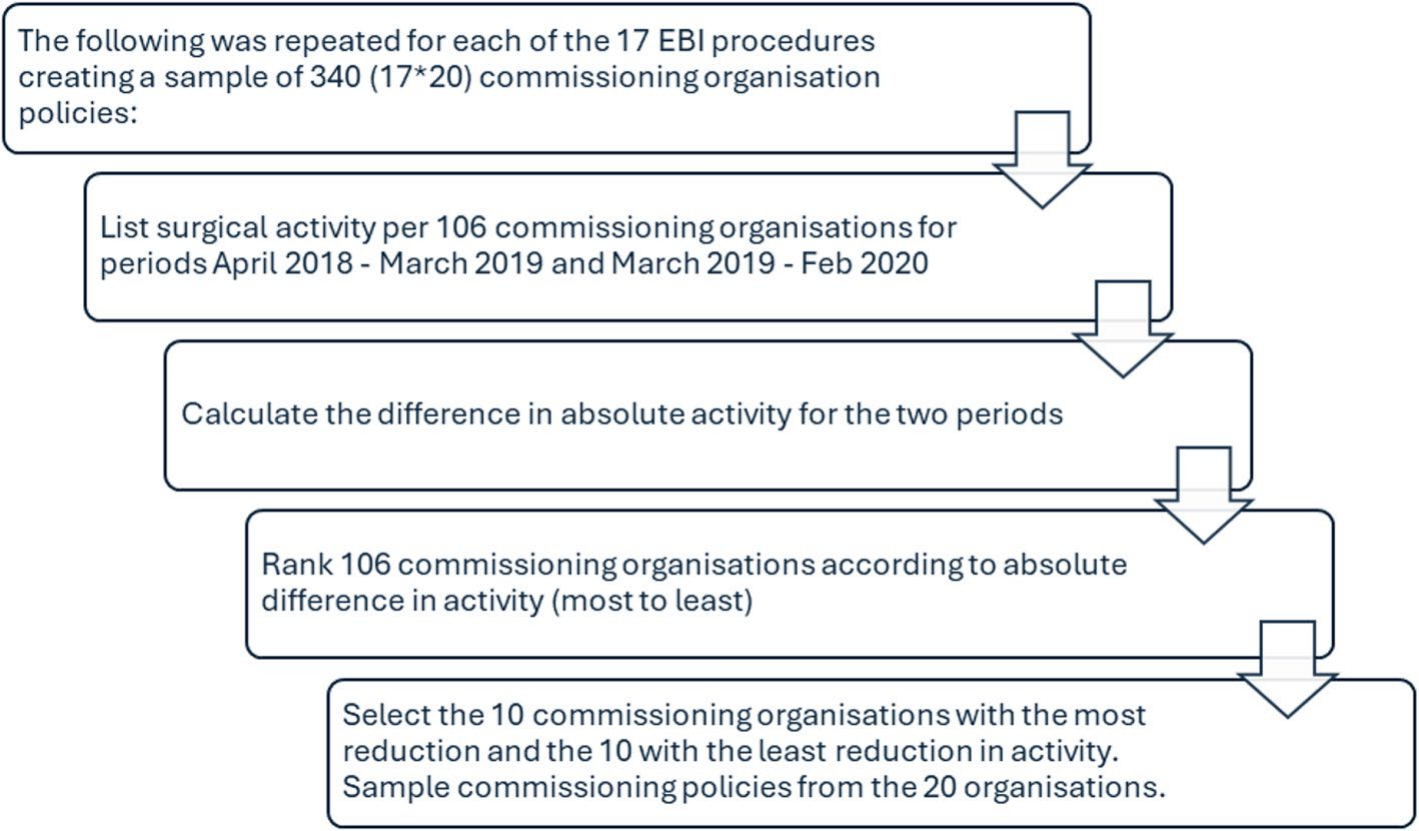
Process for sampling commissioning organisations and commissioning policies

Having identified a sample of commissioning organisations for each of the 17 EBI procedures, it was estimated that 340 commissioning policies would be sought. This was based on the assumption of 20 policies from the 20 commissioning organisations per each of the EBI procedures (no.17), being found. Figure 1 visualises the sampling process.

#### Inclusion/exclusion criteria

Commissioning policies were eligible for inclusion if they referred to the surgical procedure of interest. Policies were excluded where (1) they pre-dated publication of EBI Guidance [21], (2) they were not dated, or (3) the document could not be downloaded.

#### National EBI recommendations and local commissioning policies

EBI recommendations for each of the 17 procedures were downloaded from the Academy of Medical Royal Colleges website during the period October 2021 – November 2021 [29].

For each of the 17 surgical procedures, websites of the 20 sampled commissioning organisations were searched by researchers (NF & CC) during the period 10 August 2021–31 October 2021. Policy searches for a given procedure were conducted over the course of the same day, to ensure policies from different organisations were all ‘live’ at a consistent date. Policies were saved in pdf format for analysis.

###  Extracting data and categorisation

A table template was devised for data extraction and categorisation of commissioning organisation policies for each procedure (Additional file 1). The first table section focussed on local commissioning policy details and funding mechanism data, including: publication date, review date (if specified), and funding approval mechanism for the policy (Individual Funding Request, Criteria Based Access, Prior Approval or unknown). Completion involved recording extracted data in the table.

The second section was pre-populated with the EBI threshold criteria for the procedure. For each local commissioning policy criterion, an assessment was made and recorded as to whether it was: the ‘same’ as EBI criteria (where the wording was identical); ‘similar, same intent’ where the wording varied but was semantically the same; or ‘different’– where the subject matter of the criterion was the same but there was a difference in semantic meaning. There was an ‘additional notes’ row to capture any observations.

The third section was only completed where a local commissioning policy criterion was categorised as ‘different’. The difference was categorised according to the coding framework developed as part of a previous study by Rooshenas et al. [30]. This consisted of five pre-defined categories: ‘differences in requirements around non-surgical treatment/management’; ‘differences in required time spent using non-surgical approaches’; ‘differences in diagnostic requirements’; ‘differences in specification of symptom severity and disease progression’ and ‘threshold modifiers’ - inclusion of words such as ‘and’ or ‘or’ which had the effect of relaxing or restricting access to a surgical procedure. A verbatim extract from the commissioning policy was recorded to illustrate how the policy differed from the EBI recommendation. Whilst each criterion was only coded to a single category, some commissioning policies contained multiple criteria that differed, and this led to some policies being coded to more than one of the five categories.

Completion of the tables was carried out by two researchers (CC and MC). To ensure consistency, a third researcher (NF) independently completed tables for two policies per procedure and met with the first author (CC) to compare data extraction and management and resolve any discrepancies. A process was in place to resolve discrepancies through consultation with another author (LR), but this was not required.

### Data analysis

For each surgical procedure, descriptive statistics were used to report the percentage of commissioning policies that concorded with EBI recommendations - defined as all individual commissioning criteria using the ‘same’ or ‘similar’ wording to the EBI criteria. Secondly, frequencies of commissioning policies assigned to each of the 5 categories of difference– where one or more individual commissioning criteria differed. Thirdly, the frequency of different funding approval mechanisms per procedure, was calculated. Written summaries were made of the nature of the differences that arose between the EBI recommendations and the commissioning policy criteria for each local policy analysed. Data analysis was undertaken by CC. The emerging findings were regularly discussed by the research team and at a Study Management Group Meeting in November 2022 and October 2023.

## Results

### Identified policies

**Fig. 2:**
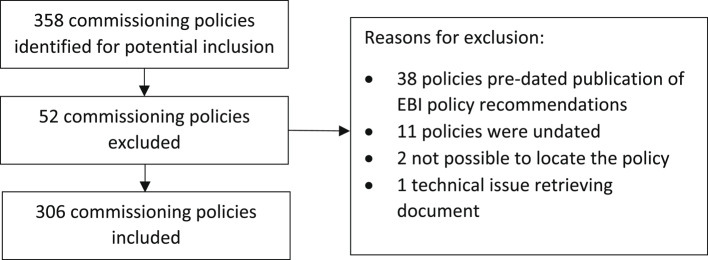
Policies identified, included and excluded in the study

A total of 358 commissioning policies were identified for potential inclusion. This was higher than expected as some commissioning organisations had more than one relevant policy. This occurred where several commissioning organisations had merged into a new organisation, but former policies were still in place. Fifty-two policies were excluded from the analysis, for reasons outlined in Fig. 2 (see Additional file 2 for procedure level detail). After exclusions, 306 (86%) commissioning polices were analysed as detailed in Table 1.

**Table 1:** Analysed commissioning policies for EBI list 1 policies

17 Surgical Procedures	No. of commissioning policies identified	No. commissioning policies excluded from analysis	No. commissioning policies analysed	Commissioning policies grouped by activity grouping	
				‘Most activity reduction’ group	‘Least activity reduction’ group
Category 1 ‘do not do’ procedures					
Snoring surgery (in the absence of obstructive sleep apnoea)	20	1	19	10	9
Dilation & curettage for heavy menstrual bleeding	20	6	14	7	7
Knee arthroscopy for patients with osteoarthritis	20	5	15	10	5
Injection for non-specific lower back pain without sciatica	21	8	13	8	5
* Category 1 Totals*	*81*	*20*	*61*	*35*	*26*
Category 2 ‘restricted access’ procedures					
Breast reduction	21	2	19	8	11
Removal of benign skin lesions	22	2	20	11	9
Grommets for glue ear in children	24	4	20	11	9
Tonsillectomy for recurrent tonsillitis	21	1	20	9	11
Haemorrhoid surgery	20	4	16	7	9
Hysterectomy for heavy menstrual bleeding	19	4	15	8	7
Chalazia removal	24	1	23	14	9
Arthroscopic shoulder decompression for subacromial pain	20	2	18	8	10
Carpal tunnel syndrome release	21	3	18	9	9
Dupuytren’s contracture release in adults	20	0	20	9	11
Ganglion excision	23	4	19	12	7
Trigger finger release in adults	21	3	18	10	8
Varicose vein interventions	21	2	19	8	11
* Category 2 Totals*	*277*	*32*	*245*	*124*	*121*
All List 1 Interventions Total	358	52	306	159	147

To reflect the imbalance in the number of procedures in Category 1 (4 ‘do not do’) and Category 2 (13 ‘restricted access’) the findings are presented separately.

### Concordance between local commissioning policies and EBI guidance

Most commissioning policies for Category 1 procedures matched EBI recommendations (72%, 44/61; Table 2). The match rate varied by surgical procedure, ranging from 60% (9 of 15 knee arthroscopy policies) to 84% (16 of 19 policies for snoring surgery). Concordance for Category 2 procedures was lower, with less than half of commissioning policies (43%, 106/245) matching with EBI recommendations. This varied by surgical procedure, ranging from an 11% match rate (2 of 19 policies for breast reduction) to 63% (10 of 16 policies for haemorrhoid surgery).

**Table 2:** Number and percentage of commissioning policies that matched EBI policy recommendations, per 17 surgical procedures and grouping of commissioning organisation

17 Surgical Procedures	Number of policies analysed	Number of matching commissioning policies (and as proportion of policies analysed - %)	Number of matching commissioning policies grouped by activity groupings	
			‘Most activity reduction’ group	‘Least activity reduction’ group
Category 1				
Snoring surgery (in the absence of obstructive sleep apnoea)	19	16 (84%)	9	7
Injection for non-specific lower back pain without sciatica	14	10 (71%)	6	4
Dilation & curettage for heavy menstrual bleeding	13	9 (69%)	4	5
Knee arthroscopy for patients with osteoarthritis	15	9 (69%)	6	3
Category 1 Totals	61	44 (72%)	25	19
Category 2				
Haemorrhoid surgery	16	10 (63%)	6	4
Tonsillectomy for recurrent tonsillitis	20	12 (60%)	7	5
Ganglion excision	19	11 (58%)	6	5
Hysterectomy for heavy menstrual bleeding	15	8 (53%)	4	4
Grommets for glue ear in children	20	10 (50%)	7	3
Dupuytren’s contracture release in adults	20	9 (45%)	6	3
Trigger finger release in adults	18	8 (44%)	5	3
Chalazia removal	23	10 (43%)	6	4
Arthroscopic shoulder decompression for subacromial pain	18	7 (39%)	3	4
Varicose vein interventions	19	7 (37%)	2	5
Carpal tunnel syndrome release	18	6 (33%)	2	4
Removal of benign skin lesions	20	6 (30%)	4	2
Breast reduction	19	2 (11%)	1	1
Category 2 Totals	245	106 (43%)	59	47

Table 2 shows the number of commissioning policies that concorded with EBI recommendations grouped according to commissioning organisations with the ‘most activity reduction’ and those with the ‘least activity reduction’. For Category 1, concordance of policies was comparable for the two groups of commissioning organisations: 73% (19/26) for the ‘least activity reduction’ group and 71% (25/35) for the ‘most activity reduction’ group. However, for Category 2 policies there was greater policy concordance amongst the ‘most activity reduction’ (48%, 59/124) compared to policies of commissioning organisations in the ‘least activity reduction’ (39%, 47/121).

### The nature of differences in commissioning policies compared to EBI policy recommendations

156 of 306 (51%) commissioning policies differed to EBI recommendations in a total of 280 categories of the five-category framework (Table 3). Full details of the differences reported for each surgical procedure are provided in Additional file 3.

**Table 3:** The nature of the differences between commissioning policies and EBI policy recommendations according to the five-category framework for classifying differences between policies

Categories of policy difference	No. of commissioning policies coded to the category of difference	Number of Category 1 surgical policies (and as a percentage of total policies that differed (17))	Number of Category 2 surgical policies (and as a percentage of total policies that differed (139))
Differences in requirement around non-surgical treatment	70	17 (100%)	53 (38%)
Differences in required time spent using non-surgical approaches	53	0	53 (38%)
Differences in diagnostic approaches	72	0	72 (52%)
Differences in specification of symptom severity and disease progression	55	0	55 (40%)
Use of threshold modifiers - and/or, ‘get-out-clauses’	30	0	30 (22%)
Total	280	17	263

All commissioning policies for Category 1 procedures included a statement that the procedures would not be routinely commissioned. Where there were differences, these were all related to the information around non-surgical treatment/management. For example, some commissioning policies for knee arthroscopy for osteoarthritis did not refer to non-surgical alternatives, such as exercise programmes, losing weight, and pain management.

139 of 245 (57%) Category 2 commissioning policies differed to EBI recommendations in a total of 263 categories of the five-category framework (Table 3). Variations were most commonly categorised as *difference in diagnostic approach* (72 of 139; 52%). For example, in some commissioning policies for grommets for glue ear, the EBI recommended criterion that children should have a specialist audiology and ENT assessment was missing. 40% (55/139) of policies contained *differences in specification of symptom severity and disease progression*. Some commissioning policies for Dupuytren’s contracture release, for example, included an additional criterion concerning the speed of progression. We also commonly observed *differences in requirements around non-surgical treatment (38%*,* 53/139); differences in required time spent using non-surgical approaches (38%*,* 53/139 policies);* and *threshold modifiers (22%*,* 30/139)*.

Commissioning policies for Category 2 procedures deviated from EBI recommendations on several fronts (Table 4).

**Table 4:** Examples of differences in commissioning policies compared to EBI policies

Examples of *differences in diagnostic requirements*
• Grommets: six local policies did not require children to have had a specialist audiology and ENT assessment and six did not include additional criterion concerning children who could not undergo a standard assessment.
• Breast reduction surgery: three local policies included lower Body Mass Index measure (BMI) cut-offs and two differed in terms of the period when the BMI needed to be stable with one having a shorter period and one a longer period.
• Benign skin lesions: eight local policies did not qualify facial spider naevi in children needing to cause significant psychological impact.
Examples of *differences in specification of symptom severity and disease progression*
• Dupuytren’s contracture release: four local polices referred to “thumb contractures” rather than “severe thumb contractures.” and five included a criterion addressing “rapid progress” of the condition over a number of months.
• Benign skin lesions: 10 local policies did not include the criterion “if left untreated, more invasive intervention would be required for removal” and eight policies did not include the criterion “The lesion causes pressure symptoms e.g. on nerve or tissue.”
• Ganglion excision: eight local policies qualified the nature of pain that needed to be experienced by referring to ‘significant’ ‘disabling’ or ‘severe’ pain.
Examples of *differences in requirements around non-surgical treatment/management*
• Arthroscopic shoulder decompression for subacromial pain: seven local polices referred to additional non-surgical treatments that needed to be tried before a referral to secondary care and four stated that surgery was not routinely funded, akin to a ‘do not do’ procedure.
• Trigger finger: six commissioning policies referred to other non-surgical treatment and two policies stipulated that non-surgical treatment include steroid injections.
• Breast reduction surgery: nine commissioning policies did not refer to the patient needing to have received a full package of supportive care from their GP such as advice on weight loss and managing pain.
Examples of *differences in required time spent using non-surgical approaches*
• Carpel Tunnel Syndrome: eight local policies required a longer period of non-surgical management ranging from ‘up to 12 weeks’ to six months.
• Chalazia management: eight local policies omitted a duration for non-surgical treatment and one stated a longer time period.
• Trigger finger: three local policies referred to a longer period for attempting non-surgical management ranging from four to six months and three local policies did not specify a time period.
Examples of commissioning policies containing *threshold modifiers*
• Hysterectomy for heavy menstrual bleeding: four local polices referred to the same sequential approach to treatments but inserted ‘and’ between each with the effect that a patient needed to have tried more treatments before consideration of a hysterectomy.
• Carpel tunnel syndrome: six local polices included the word “and” rather than “or” with the effect that the patient needed to have been unsuccessfully treated with a steroid injection and night splints to be considered for a surgical procedure.

### Funding approval mechanisms

**Fig. 3:**
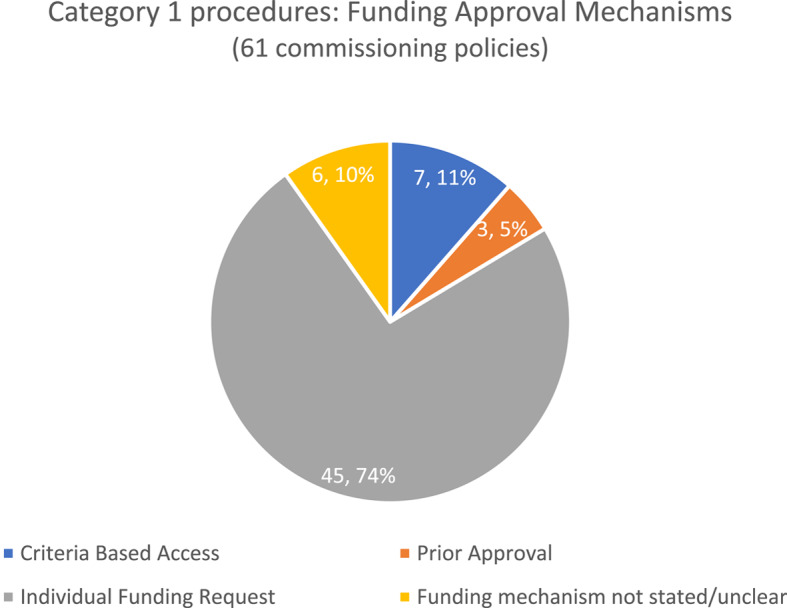
Funding approval mechanisms stated in commissioning policies: Category 1 Procedures

The funding approval mechanism most frequently stated in commissioning policies for Category 1 procedures was an Individual Funding Request (74%, 45/61; Fig. 3) as recommended by the EBI programme. However, some policies used Criteria Based Access 11% (7/61) or Prior Approval (5% (3/61)). The funding mechanism was not stated or unclear in the remaining 10% of policies (6/61).

**Fig. 4:**
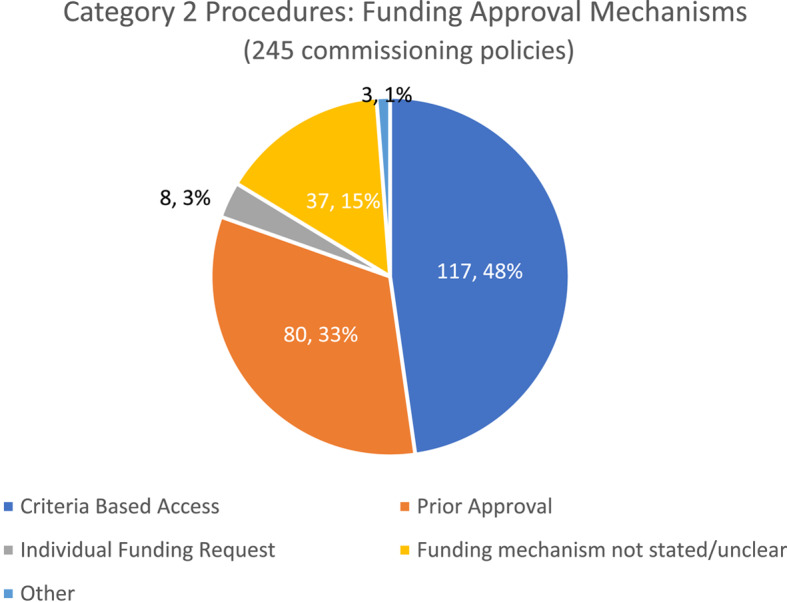
Funding approval mechanisms stated in commissioning policies: Category 2 Procedures

Commissioning policies for Category 2 procedures tended to use either Criteria Based Access (48%, 117/245) or Prior Approval (33%, 80/245) funding mechanisms (Fig. 4). Considerably fewer policies required an Individual Funding Request (3% (8/245)). The funding mechanism was unclear or not stated in 15% (37/245)) of policies.

Considered at a procedure level (Table 5), there was much variation in the use of funding mechanisms. Reference to Criteria Based Access ranged from 11% (breast reduction 2/19) to 74% (ganglion excision (14/19) whilst use of Prior Approval ranged from 15% (grommets for glue ear in children (3/20) to 53% (breast reduction (10/19).

**Table 5:** Funding approval mechanisms used by commissioning organisations to manage category 2 procedures

Category 2 Surgical Procedures	Number of policies analysed	Number of policies stating Criteria Based Access (CBA) (and as proportion of policies analysed - %)	Number of policies stating Prior Approval (PA) (and as proportion of policies analysed - %)	Number of policies stating Individual Funding Request (IFR) (and as proportion of policies analysed - %)	No. of policies where mechanism not stated/unclear (and as proportion of policies analysed - %)	Other (and as proportion of policies analysed - %)
		Total	Total	Total	Total	Total
Ganglion excision	19	14 (74%)	5 (26%)	0	0	0
Carpal tunnel syndrome release	18	13 (72%)	3 (17%)	0	2 (11%)	0
Chalazia removal	23	14 (61%)	7 (30%)	0	2 (9%)	0
Varicose vein interventions	19	11 (58%)	5 (26%)	1 (5%)	2 (11%)	0
Haemorrhoid surgery	16	9 (56%)	4 (25%)	0	2 (13%)	1 (6%)
Trigger finger release in adults	18	10 (56%)	6 (33%)	0	2 (11%)	0
Grommets for glue ear in children	20	10 (50%)	3 (15%)	0	6 (30%)	1 (5%)
Hysterectomy for heavy menstrual bleeding	15	7 (47%)	6 (40%)	0	2 (13%)	0
Dupuytren’s contracture release in adults	20	9 (45%)	5 (25%)	0	6 (30%)	0
Removal of benign skin lesions	20	7 (35%)	10 (50%)	0	3 (15%)	0
Tonsillectomy for recurrent tonsillitis	20	7 (35%)	9 (45%)	0	3 (15%0	1 (5%)
Arthroscopic shoulder decompression for subacromial pain	18	4 (22%)	7 (39%)	3 (17%)	4 (22%)	0
Breast reduction	19	2 (11%)	10 (53%)	4 (21%)	3 (16%)	0
Category 2 Totals	245	117	80	8	37	3

The use of funding approval mechanisms by commissioning organisation was compared for those in the ‘most activity reduction’ group and those in the ‘least activity reduction’ group (Supplementary Material (SM4)). For Category 1 procedures, there was little variation in use of funding mechanism between the two groups. Whilst for Category 2 procedures, where stated (205 policies), the dominant funding mechanism for both groups was Criteria Based Access. Commissioning organisations in the ‘most activity reduction’ group however, used it more (61% 60/98) compared to commissioning organisations in the ‘least activity reduction’ group (53% 57/107). Conversely, Prior Approval featured more in policies of commissioning organisations in the ‘least activity reduction’ group (43% 46/107) compared to those in the ‘most activity reduction’ group at 35% (34/98).

## Discussion

Despite the launch of a national programme to promote standardised de-adoption policy, our study found that just under half (49%) of sampled local commissioning policies analysed concorded with these recommendations. Where local policy criteria differed to national policy, the differences were variable, but most commonly categorised as *differences in diagnostic approach*. Whilst commissioning organisations largely followed a uniform approach to funding mechanisms to manage requests for ‘do not do’ procedures, the picture was more mixed for ‘restricted access’ procedures, where ‘Criteria Based Access’ was used in nearly half of policies and ‘Prior Approval’ in a third.

The EBI Guidance acknowledged that there was considerable variation in rates of interventions across England [21]. Studies have found geographical variation in policies managing patient access for surgical treatment (whether or not it has EBI recommendations for de-adoption) including for: carpal tunnel [31], varicose veins [32], breast surgery [33], musculoskeletal procedures [30, 34], bariatric surgery [35] and hand conditions [36]. Typically, these studies concluded that greater consistency across commissioning policies was needed to achieve more uniform surgical activity and standards of care. To achieve such standardisation, Rooshenas et al. [30], recommended that greater uniformity might be achieved if national bodies lead on articulating criteria for accessing treatment. The findings here suggest that the publication of guidance by a national body alone is not enough to secure standardised policy.

The limited compliance between national and local policies illuminated in this study may provide some explanation for why the EBI programme was observed to have limited effect on reducing surgical activity and geographic variation. This was shown via two independent evaluations that quantitatively examined the effect of the EBI programme on surgical activity rates [10, 11] for the 17 procedures that were the subject of this study. The study by Anderson et al. [10] included a comparison of activity between commissioning organisations who had trialled the EBI recommendations before the programme launch, a group referred to as the ‘demonstrator community,’ and those that had not, and found no significant difference in activity. Furthermore, the study by Glynn et al. [11] found no evidence of change in geographical disparities in surgical activity for these 17 procedures.

There is a considerable body of evidence to show that implementation of (or adherence to) any new programme– including de-adoption programmes - is more likely if multiple interventions are employed rather than producing guidance alone [19, 37]. Reference to the EBI programme in the NHS Standard Contract and introduction of the zero-tariff rule for non-compliance could be viewed as two levers to support adherence, although our findings indicate otherwise. One interpretation of this is that the statutory status of the EBI programme was not a sufficient mechanism for compliance. It is possible that mixed communications relating to the statutory nature of the EBI programme diluted messaging about the extent to which adherence was mandatory. The EBI Guidance contained an extract from the NHS Standard Contract Consultation Draft 2019/20 [25] which allowed local commissioning policy to vary where local policy was more stringent. This draft clause was removed from the NHS Final Contract 2019/20 [38], the explanation being ‘to promote consistency’ [39]. It is possible that the changes to the final NHS contract may account for some non-compliance we observed. Some commissioning policies included in our analysis stated that they were not adopting the national recommendations because these would widen access to the procedure, thus exerting cost pressures. Another strand of the OLIVIA study, examines commissioners’ accounts of how they responded to the EBI programme [40].

Other interventions to promote compliance with the EBI Guidance included the establishment of ‘demonstrator communities’– groups of commissioning organisations, engagement with clinicians and specialist societies, and production of patient facing videos and written information. Whilst clearly a multi component approach, perhaps more active interventions are needed to secure local adoption of national guidance.

In designing this study, there was an implicit assumption that standardisation of written policies matter, because they have implications for practice. However, previous research has shown that clinicians’ practices often do not adhere to national or local policies [33, 35, 41]. Russell and colleagues [33], for example, acknowledge the ‘intuitive appeal’ of consistent policies, in their study examining Individual Funding Requests for breast surgery, but concluded that the interpretative and contextual nature of policy implementation made the standardisation of policy an ‘illusory policy goal’. Clinicians’ response to the EBI programme and in particular, the national recommendations were investigated as part of the OLIVIA study and will be the subject of a forthcoming publication.

The facilitative role that funding mechanisms can have in securing de-adoption is identified in literature reviews by Elshaug et al. [13] and Niven et al. [42]. It is not surprising that varying funding mechanisms for ‘restricted access’ procedures were identified, given that the EBI programme left these decisions to the discretion of local commissioning organisations. While it is possible that enforcing stricter funding mechanisms universally might have supported de-adoption (i.e. reduced provision) of the procedures, previous research has shown that prior approval mechanisms can be resource-intensive for commissioners [41, 43]. Choice of funding mechanism is thus a trade-off between resource considerations and their effectiveness in enhancing adherence to policies.

### Strengths and limitations

This study, to our knowledge, is the first to investigate the effectiveness of national policy guidance as an intervention for achieving standardised local de-adoption policies. Furthermore, this is the first study to examine a sample of commissioning policies across a full national de-adoption programme.

This study provides a snapshot of analysis at a point in time and as such it is not possible to comment on what changes, if any, commissioning organisations made to comply with EBI recommendations. We considered mapping out what changes had been made to the policies we examined by searching their ‘version history’ sections, but these were not consistently available and varied in the extent of detail they provided. We relied on publicly available commissioning policy documents and whilst contact with commissioning organisations and Freedom of Information requests might have yielded more policies and information, it was not feasible within the time frame of this study. It is also possible that the research was conducted too soon, leaving commissioning organisations insufficient time to change their policies.

The decision to sample a proportion of commissioning policies per surgical procedure was a practical one, given that analyses were being conducted for 17 procedures. Capturing all commissioning policies, for every surgical procedure, would have yielded a more comprehensive assessment of compliance with the EBI programme, but this was not feasible within the timescales of this study.

### Implication

The implication of this study for future national level de-adoption initiatives is that the publication of national de-adoption guidance needs to be accompanied by linked interventions to actively promote and support local commissioning organisations to reflect national policy.

### Future research

Unlike implementation of new services/technologies, de-adoption concerns entrenched practices, embedded in often localised decision-making structures, policies, and processes. Building on this study, there is a need for future research to investigate what support devolved healthcare purchasing bodies (e.g. commissioners) need to facilitate compliance with national de-adoption policy. There is also value in examining what factors are associated with ‘compliant’ and ‘non-compliant’ devolved bodies, and how these relate to activity rates for the health technology (e.g. surgical procedure) in question.

## Conclusion

While previous research has called for national de-adoption policies, our study showed that production of these recommendations does not universally translate into compliance at local level - even if national recommendations are statutory. More support is needed for devolved health care purchasers to comply with national guidance.

## Supplementary Information

Supplementary Material 1.

Supplementary Material 2.

Supplementary Material 3.

Supplementary Material 4.

## Data Availability

The datasets used and/or analysed during the current study are available from the corresponding author on reasonable request.

## Abbreviations

CBA: Criteria Based Access (CBA)
EBI: Evidence-Based Interventions
HCP: Healthcare Professional
ICB: Integrated Care Boards
IFR: Individual Funding Request
NHS: National Health Service
NICE: National Institute for Health and Care Excellence
PA: Prior Approval
